# Polymerase θ Coordinates Multiple Intrinsic Enzymatic Activities during DNA Repair

**DOI:** 10.3390/genes12091310

**Published:** 2021-08-25

**Authors:** Karl E. Zahn, Ryan B. Jensen

**Affiliations:** 1 Department of Therapeutic Radiology, Yale University School of Medicine, New Haven, CT 06520, USA; 2 Repare Therapeutics, 7210 Rue Frederick Banting, Montreal, QC H4S 2A1, Canada

**Keywords:** DNA repair, synthetic lethality, *POLQ*, polymerase θ, cancer, PARP-i, polθ-i, 5′-end resection, shieldin, *53BP1* effector

## Abstract

The *POLQ* gene encodes DNA polymerase θ, a 2590 amino acid protein product harboring DNA-dependent ATPase, template-dependent DNA polymerase, dNTP-dependent endonuclease, and 5′–dRP lyase functions. Polymerase θ participates at an essential step of a DNA double-strand break repair pathway able to join 5′-resected substrates by locating and pairing microhomologies present in 3′-overhanging single-stranded tails, cleaving the extraneous 3′-DNA by dNTP-dependent end-processing, before extending the nascent 3′ end from the microhomology annealing site. Metazoans require polymerase θ for full resistance to DNA double-strand break inducing agents but can survive knockout of the *POLQ* gene. Cancer cells with compromised homologous recombination, or other DNA repair defects, over-utilize end-joining by polymerase θ and often over-express the *POLQ* gene. This dependency points to polymerase θ as an ideal drug target candidate and multiple drug-development programs are now preparing to enter clinical trials with small-molecule inhibitors. Specific inhibitors of polymerase θ would not only be predicted to treat BRCA-mutant cancers, but could thwart accumulated resistance to current standard-of-care cancer therapies and overcome PARP-inhibitor resistance in patients. This article will discuss synthetic lethal strategies targeting polymerase θ in DNA damage-response-deficient cancers and summarize data, describing molecular structures and enzymatic functions.

## 1. Introduction

Faithful segregation of chromosomes by equal sharing of genetic material between daughter cells is critical for genomic stability. Signaling cascades known as checkpoints evolved to arrest the cell cycle in response to DNA lesions capable of skewing the distribution of inherited genetic material [1]. Checkpoint activation is an essential component of the DNA damage response (DDR), ensuring that DNA repair machinery has time to restore DNA before chromosomes are subjected to the physical stresses of cell division. Animals with defects in genes coding for DNA repair enzymes or other DDR proteins may survive with elevated rates of genomic instability. However, given enough time or sufficient exposure to environmental mutagens, DDR mutants will disproportionately succumb to genetic diseases such as cancer. The field of precision oncology looks to exploit weaknesses inherent in cancer cells by testing the hypothesis that specific genetic backgrounds underpin cancer predisposition or fortify the tumor microenvironment, and are therefore treatable by likewise specific and targeted therapies [2,3]. This review will explore the enzymatic functions of DNA polymerase θ (pol θ) that will soon be targeted by small-molecule inhibitors in cancer therapy clinics for the first time, with hopes that patients may benefit from this synthetic lethal approach.

## 2. DNA Double-Strand Break Repair in Mammalian Cells

**Figure 1:**
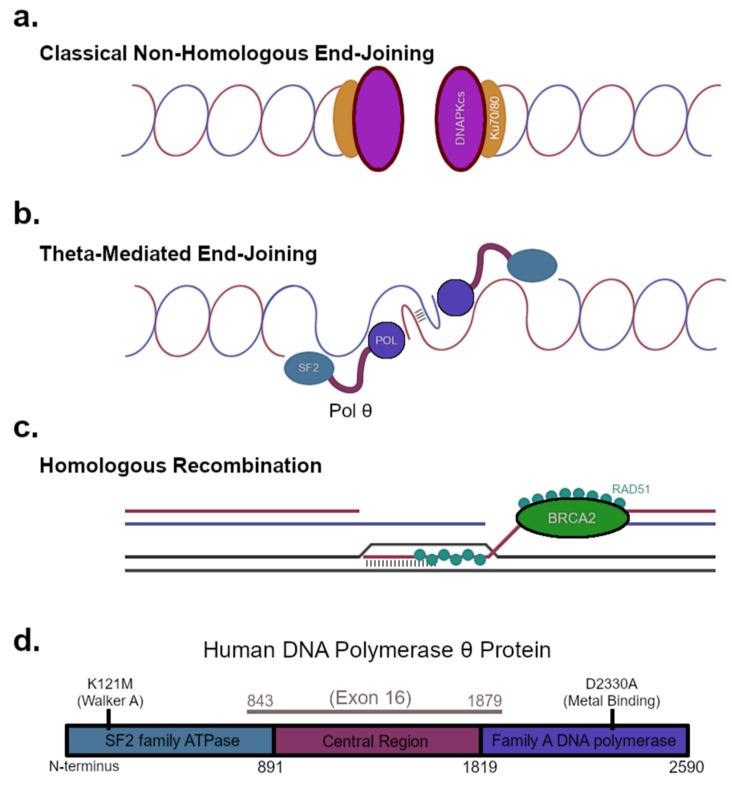
DNA DSBs are repaired by (**a**) C-NHEJ, (**b**) TMEJ (SF2 = super-family II ATPase domain; POL = Family A DNA polymerase domain), and (**c**) HR. (**d**) The protein architecture of the *POLQ* gene product is shown with the locations of inactivating mutations in the DNA-dependent ATPase (K121M) domain and template-dependent DNA polymerase (D2330A) domain labeled.

DNA double-stranded breaks (DSBs, Figure 1) present a severely toxic lesion because shearing of chromosomes becomes likely if opposing knicks are present in the DNA phosphate backbone. Homologous recombination (HR) utilizes a sister chromatid for template-directed repair of DNA DSBs with low rates of mutation and may have evolved as a means of replication fork restart [4]. Canonical HR is initiated by a 5′-end resection at the DSB by the MRN nuclease complex. Additional nuclease complexes comprised of BLM helicase and DNA2 or EXO1 [5] catalyze long-range end resection, yielding extended 3′-overhanging DNA rapidly coated with RPA. Mediator proteins such as BRCA1 [6] and BRCA2 [7] serve a critical role exchanging RPA for RAD51 recombinase onto ssDNA 3′-overhangs (Figure 1c). BRCA1 also regulates the first steps of the 5′-end resection [8]. The RAD51-ssDNA filament then initiates a homology search, forming a displacement-loop (D-loop) as it invades the sister chromatid. The D-loop establishes a primed 3′-end for extension by DNA polymerases, followed by second-end capture, Holiday junction resolution and ligation.

An essential option for DSB repair is available to cells when a replicated chromatid is not present to direct the repair process [9]. Classical non-homologous end-joining (C-NHEJ) is regulated in part by *53BP1*, which blocks 5′-end resection until CtIP becomes phosphorylated by cyclin-dependent kinases (CDKs) and ATM [10] to promote MRN nuclease-complex function. When Ku70/Ku80 retains the DSB-ends and recruits DNA-PKcs (Figure 1a), C-NHEJ factors assemble a DNA repair complex capable of directly ligating the DSB by the catalytic activity provided by *LIG4*. This process requires little, if any, DNA synthesis and is active not only during G1, but throughout the cell cycle. Polymerases λ and μ synthesize DNA opposite a discontinuous template strand to establish ligatable sticky ends [11]. In principle, C-NHEJ can mutate DNA sequences, especially if chemically blocked ends obstruct ligation of the DSB or extension of a 3′-terminus. Despite this inherent challenge, C-NHEJ does not require extensive end-resection, provides exquisite fidelity when executed in an appropriate DNA repair context, and therefore, serves as the front-line DSB repair pathway in eukaryotic cells [12]. 

Cells maintain a balance of DSB repair pathway choices to avoid deleterious events. Mice with homozygous exon 11 truncations in *BRCA1* (*BRCA1^d11^*) abort during development. Concomitant inactivation of *53BP1* reverts this developmental defect [13] by restoring DSB-break repair by HR [14,15] and these tandem genetic defects allow the double-mutant pups to live extended life spans. The *53BP1* attenuating mutation S25A deletes a key ATM phosphorylation site required for interactions with PTIP. 53BP1 normally binds PTIP on chromatin to block end-resection via this key phosphorylation-dependent interaction [16]. The *53BP1^S25A^* allele is permissive to end-resection by *Dna2* and thereby rescues embryonic development of *BRCA1^d11^* mice. RIF1 and the shieldin complex (*Shld1-3* and *Rev7*) [17] serve as additional effectors of *53bp1*, which retain function in the context of *53BP1^S25A^*. *BRCA1^d11^* mice rescued by *53BP1^S25A^* therefore exhibit an HR-insufficient phenotype, due to RIF1/shieldin-limited end-resection, leading to premature aging and death [18]. These seminal rescue experiments provide a wide view of how HR and C-NHEJ—as the predominant mechanisms of DSB repair in mammalian cells—must cooperate to maximize genomic stability. Cells regulate 5′-end resection as the primary mechanism enforcing this balance.

When C-NHEJ is impaired by *LIG4* knockout, studies have shown that backup end-joining mechanisms persist to engage in DSB repair [19]. These alternative repair pathways have been referred to as microhomology mediated end-joining (MMEJ, or alternative EJ) to describe the repair signature left at sites of DNA repair. *LIG3* was deemed a critical MMEJ repair factor in early experiments that were otherwise without unifying genetic determinants [20]. More recently, *POLQ*, the gene coding for pol θ, has been attributed a defining role in this backup mode of DSB repair. Theta-mediated end-joining (TMEJ) [21] accounts for a major portion of the MMEJ signatures arising as genetic scars of DSB repair (Figure 1b). TMEJ (and not C-NHEJ) is likely responsible for large deletions and indel mutations at Cas9-targeted DSBs in mouse embryonic stem cells [22]. Advances in CRISPR-Cas9 technology as a screening tool to identify synthetic lethal genetic interactions has expanded the capabilities of researchers to evaluate compensatory mechanisms activated in DSB repair mutant backgrounds. Pol θ, though non-essential in most wildtype backgrounds, assumes a critical role for repair of DSB repair once either the HR or NHEJ apparatuses are attenuated [23,24,25]. This holds in patient derived human cell lines harboring low-activity *BRCA1*, or especially *BRCA2* alleles [26,27]. Some alleles with high frequency in humans manifest as exon 11 deletions due to splice-site mutations in *BRCA1* or the 6174delT premature stop truncation of *BRCA2* [28]. Although substantial progress has been made validating pol θ as an ideal drug target, the precise molecular mechanism by which TMEJ diverts catastrophic genomic instability in many mutant DNA repair backgrounds persists as an intriguing question. Pol θ is a large multifunctional DNA repair enzyme with a DNA-dependent ATPase at its N-terminus, sharing homology to super-family II (SF2) helicases, a poorly conserved central region with enigmatic functions, and a family A DNA polymerase at its C-terminus sharing homology with Klenow Fragment and Taq polymerases (Figure 1d). What would constitute the most efficacious mechanism of action for small-molecule inhibitors? Researchers will require additional structural data to understand how the multiple domains cooperate to achieve DNA repair. The field also lacks an expansive in vitro reconstitution assay that incorporates the numerous steps of TMEJ. These omissions constitute a large gap in knowledge that limits rational drug development and warrants additional effort to understand the structure-function relationships that uniquely arm pol θ to protect the genome when front-line DNA repair mechanisms go awry.

## 3. Synthetic Lethal Interactions with Checkpoint and DNA Repair Genes

The chaos1 allele of *POLQ* originated in a forward genetics screen in mice treated with *N*–ethyl–*N*–nitrosourea (ENU), a powerful germline mutagen [29]. Mice homozygous for *POLQ^chaos1^*, though viable and phenotypically normal in laboratory settings, accumulate micronucleated erythrocytes detectable in blood by propidium iodide staining. *ATM* kinase serves as an essential regulator of the checkpoints activated in response to DNA DSBs. Crosses of *POLQ^chaos1^* with *ATM*-deficient mice deviate from expected Mendelian ratios, suggestive of a deleterious genetic interaction stemming from defective DSB repair. Additional synthetic lethal interactions with *POLQ* were identified in mice deficient of *FANCD2* [23]. The FANCD2 protein operates at the interface of the Fanconi anemia pathway and canonical *BRCA2*-mediated homologous recombination (HR). Unlike *BRCA2*, *FANCD2* is non-essential in mice, although knockout animals often develop tumors [30] and suffer hematopoietic defects [31]. Conditional knockout of *BRCA1* or *BRCA2* in MEF cells are known to induce radial and broken chromosomes in conjunction with shRNA targeting *POLQ* [24]. Furthermore, *POLQ*-knockout MEF cells are sensitive to DSB-inducing agents, such as topoisomerase inhibitors and ionizing radiation [32]. In addition to recombination-repair factors, synthetic sick phenotypes have been reported for MEF cells in which *Ku70* was co-depleted with *POLQ* [25]. These studies corroborate TMEJ as a backup DSB repair mechanism essential for viability in DSB-repair-deficient backgrounds. A targeted DDR CRISPR screen completed in *POLQ*-deficient MEF cells expanded the breadth of synthetic lethal pairs to include a wide range of genes participating in multiple mechanisms of DNA repair, especially base excision repair (BER). This strategy identified additional HR, C-NHEJ, and DDR signaling factors [33]. The screen also confirmed prior findings that *53BP1* is synthetic lethal with *POLQ* in MEF cells, suggesting that unviability derives from the accumulation of abortive HR intermediates during S-phase, rather than impairment of C-NHEJ directly. Collectively, studies employing mouse genetics and mouse-derived cell lines have validated *POLQ* as a critical gene maintaining genomic integrity in multiple DNA-repair-deficient backgrounds. DNA lesions of varied origin eventually give rise to DSBs, if left unrepaired. This explains why the TMEJ pathway compensates for multiple forms of persistent DNA damage and/or replication stress.

## 4. Enzymatic Activities Coordinated by Pol θ

At 2590 amino acids, the physical size of pol θ—not to mention its relative scarcity in cells—presents numerous technical obstacles to in vitro study. Biochemical characterization of pol θ began by the cloning of a full-length cDNA in 2003 [34]. Sequence homology to SF2 helicases in the 5′-region and family A DNA polymerases in the 3′-region set the stage for incremental characterization of pol θ function [35]; however, they have yet to determine why this protein—conserved in all multicellular organisms except fungi—wields these functional domains in tandem. The central region of pol θ, though poorly conserved in terms of size and sequence across species, tethers the ATPase and pol domains together. Interestingly, a single exon spans the entire central region (Figure 1d) [34]. This physical linkage, however, does not establish *a priori* that enzymatic activities from both domains must cooperate in every given biological process.

The in vivo function of pol θ transcends DNA repair. Pol θ is known to impinge on replication timing in unstressed mammalian cells, possibly by direct interactions with origins of replication [36]. The *POLQ* homolog TEBICHI, conserved in plants, is critical for normal cell differentiation and division in *Arabidopsis thaliana* [37]. Interestingly, teb-alleles lacking the polymerase domain exhibit none of the growth defects due to the alleles truncating the ATPase domain. The studies above lack systematic complementation analyses employing ATPase dead point-variant alleles, which would be very interesting from a drug-development standpoint. However, the anti-cooperation hypotheses for varied pol θ enzymatic functions deserve consideration, given that drug-development programs are targeting the pol θ ATPase as a strategy for cancer therapies intended to primarily block TMEJ in DDR-deficient genetic backgrounds [26].

The template-dependent family A DNA polymerase domain harbors arguably the most important enzymatic activity of pol θ because inactivating mutations in the conserved polymerase motifs (Figure 1d) fails to complement DSB sensitivity in cell lines derived from *POLQ*-knockout mice [32]. Inactivation of the ATPase domain by mutation of the Walker A nucleotide-binding motif (Figure 1d) impacts the sequence of repair products [25,38] and may not be tolerated in certain human cancer cell lines [23], but ATPase dead mutants strikingly complement DSB-intolerance in knockout MEF cells [32]. In fruit flies and flat worms, orthologs of pol θ repair interstrand-crosslinks (ICLs) [39] and the ATPase activity is essential for resistance to nitrogen mustard in *Drosophila melonogaster* [38]. Prior experiments with the full-length pol θ, purified from HeLa cells, convey a DNA-dependent ATPase function but fail to demonstrate helicase activity on model substrates [34]. This conundrum has been reported subsequently by several groups [40,41]. An optimized in vitro system has shown that an N-terminal fragment of pol θ may have the ability to melt certain dsDNA substrates when supplied with a complementary acceptor ssDNA strand [42], or potentially to strip RPA or RAD51 from small ssDNA overhangs [43]. The human gene product of HELQ shares 34% sequence identity with pol θ across a consecutive 770 amino acids of the N-terminal ATPase domain [44], but functional redundancy with pol θ is questionable. *HELQ* knockdown in human endocervical cancer cells renders them sensitive to MMC [45]. HELQ is known to interact directly with ATR and the RAD51 paralogs [46], excluding XRCC3 [47], although a regulatory role for HELQ in ATR-mediated checkpoint signaling via this interaction is debatable. HELQ-dependent removal of RAD51 or RAD51 paralogs from HR intermediates could assist replication-coupled DNA repair [47]. Paradoxically, HELQ accomplishes a DNA-cleaning function in vitro independent of the catalytic ATPase activity, when provided with RAD51-coated dsDNA substrates [48]. Purified ATPase proficient HELQ protein unwinds model replication fork substrates in vitro [49] without any of the issues surrounding the low specific activity of the pol θ N-terminal ATPase construct. Additional experimentation will be necessary to clarify why the pol θ ATPase lacks the strong enzymatic activity of HELQ [50], or the archaeal homolog HEL308, which can break the biotin–streptavidin interaction due to tenacious helicase and protein-stripping functions [51]. 

## 5. Structures of Template-Dependent DNA Polymerase and the DNA-Dependent ATPase

**Figure 2:**
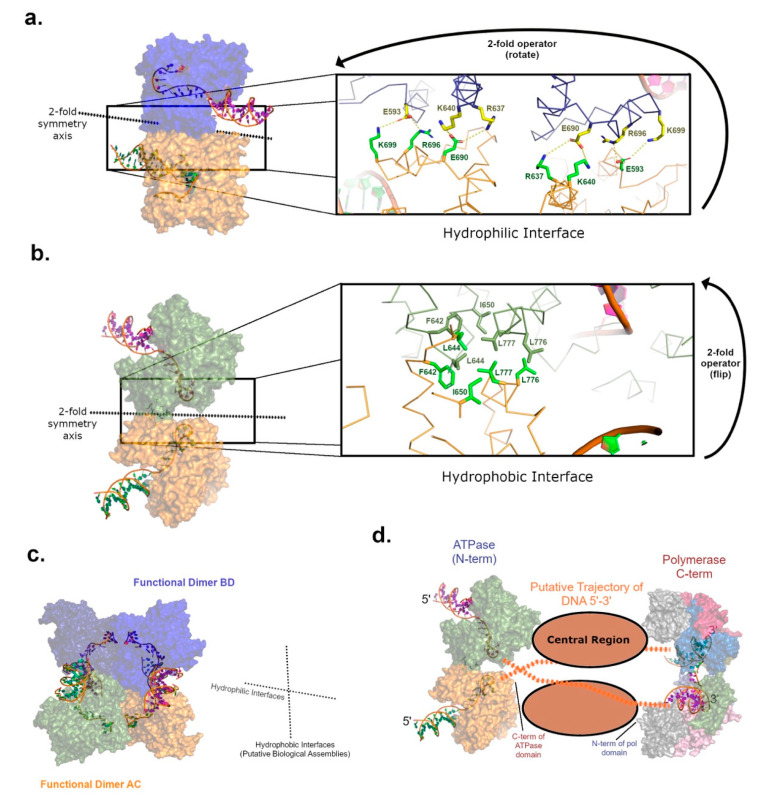
The pol θ ATPase and polymerase crystal structures provide dimer-of-dimers configurations (D2 symmetry) in independent structural investigations [40,53]. A homology model is generated by superimposing protein residues from the homologous structure of HEL308 (PDBID 2P6R) [51] onto the pol θ ATPase structure (PDBID 5A9J) to obtain proxy for the DNA binding mode. (**a**) One dimer interface of the overall pol θ ATPase structure is propagated by hydrophilic amino acids (brown and blue surface) with the capacity to form salt bridges. (**b**) The adjacent dimer interface is hydrophobic in character (brown and green surface) and further stabilized by a short region of antiparallel β-strands in the vicinity of F642. (**c**) Selecting the hydrophobic interface as an obligatory set of interactions defines the probable functional biological assembly (protein chains BD and AC interact via the vertical 2-fold axis represented by dashed lines). The hydrophilic interfaces (horizontal 2-fold axis) could dissociate at some point during catalysis of TMEJ or exist as an artifact of the protein truncation. (**d**) The striking compatibility of the predicted dimeric biological assemblies informed by the crystal structures of the pol θ ATPase and polymerase domains infers that 3′-overhanging DNA could traverse dimers of pol θ full-length from 5′ to 3′, starting at the N-terminal ATPase domain and running towards the C-terminal polymerase domain (PDBID 2X0P). The DNA could pass from the ATPase domain of one pol θ molecule to the pol domain of the other (as shown), without occluding the predicted location of the central region.

The crystal structure of the ATPase domain from pol θ (Figure 2) reveals tetrameric oligomerization as a dimer-of-dimers in two alternative crystal forms [40]. Sedimentation analysis, multi-angle light scattering coupled with size exclusion chromatography (SEC-MALS), and small-angle X-ray scattering (SAXS) corroborated this high-order configuration, which occurs independent of ATP binding. One hypothesis casts the ATPase domain as a scaffolding motif able to stabilize synaptic pol θ complexes during the end-joining of breaks [40]. Dimerization is often observed for DNA repair enzymes able to synapse DSBs, such as DNA-PK or LIG4, but functional tetramerization aligns more with helicases catalyzing branch migration of 4-way DNA junctions [52]. Like the pol θ ATPase, HELQ was also shown to exist in solution as a tetramer by SEC-MALS, but emerging evidence implies that active complexes of the HELQ could exist as dimers, hinting that the tetrameric form might serve a regulatory purpose [50]. Identifying the functional biological assembly assumed by the pol θ ATPase domain in cells warrants further analysis. 

As a dimer-of-dimers (Figure 2c), the protein–protein interfaces presented by the pol θ ATPase crystal structure manifest as two types: one interface possesses significant hydrophobic character and several potentially critical protein backbone–backbone interactions (Figure 2b) while the alternate interface is populated by likely ionic salt-bridge interactions (Figure 2a). This observation could be interpreted as support for the hypothesis that tetramerization occurs as a negative regulatory mechanism. The charged surface of the ATPase domain would readily interact with aqueous solvent, for example, once the ionic protein–protein interface was broken in response to binding the proper DNA substrate. More experimentation with the pol θ ATPase will be needed to rectify predictions for DNA helicase activity, based on amino acid sequence homology to other functional helicases, with the empirical lack of activity in vitro reported by multiple research groups.

The pol θ ATPase is known to bind DNA tightly (low nM range) [40]. Unfortunately, structural information describing the DNA binding mode is not yet available, although a homologous DNA-bound structure exists for HEL308 [51]. HEL308 does not oligomerize like HELQ or the pol θ ATPase, but does provide a reference point for homology modeling (Figure 2). When coordinates for the HEL308 DNA-bound structure are transformed onto the pol θ model, by aligning protein residues, the DNA binding mode orients the single-stranded 3′-extension of the substrate near the C-terminal end of the pol θ ATPase construct. The location of the DNA exit tunnel in the ATPase shows that ssDNA could project past the central region and into the C-terminal polymerase active site of full-length pol θ (Figure 2d). Notably, the polymerase domain ternary complex crystal structure (pol, DNA, nucleotide) also illuminates a dimeric biological assembly [53]. These high-order structures are strikingly compatible and define a plausible trajectory for a continuous DNA substrate (Figure 2d), but this hypothesis requires selection of one ATPase dimer pair out of the overall D2-symmetrical arrangement. This conclusion implies that the excluded dimer is an artifact of the protein construct or comprised of interactions that dissociate during TMEJ.

## 6. The DNA Polymerase Activity of Pol θ

**Figure 3:**
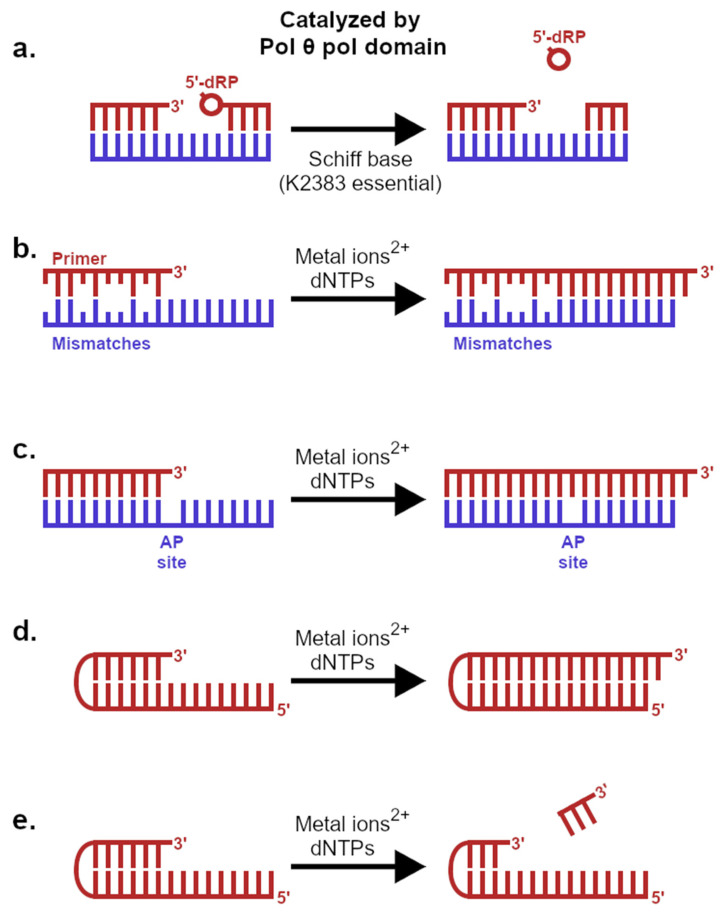
The pol domain of pol θ catalyzes multiple DNA-modifying activities. (**a**) 5′-dRP lyase activity removes ribose fragments. (**b**) Minimally primed, template-directed primer extension. Pol θ adds a single nucleotide to a blunt duplex, establishing a 3′-overhang. (**c**) Translesion primer extension opposite blocking lesions such as AP sites. (**d**) Intramolecular snapback synthesis via a hairpin structure. (**e**) Deoxy-NTP-dependent 3′-end trimming.

The polymerase domain of pol θ possess unprecedented DNA-modifying activities leading to its specialization for end-joining (Figure 3). The crystal structure of the polymerase domain revealed charged amino acids providing pol θ-specific interactions to the primer DNA strand [53], not possible in bacterial homologs such as Klenow or Taq. These interactions are mediated by the residues: R2254, R2202, and K2181. In particular, R2254 (equivalent site of Val or Ile in prokaryotic homologs) proves essential for translesion synthesis opposite a model AP site or thymine glycol (Figure 3c). The R2254V mutation provides a separation-of-function variant able to catalyze standard template-dependent DNA synthesis but unable to bypass DNA lesions, which the parental variant accomplishes readily [53]. Pol θ may bypass UV-induced DNA-lesions [54] in cells, where it is also believed to incorporate dT correctly opposite 1, N6-ethenodeoxyadenosine (ϵdA) [55]. Pol θ could provide lesion bypass activity redundant with BER [56]. *C. elegans*, as a particularly interesting example, lacks a homologous *POLB* gene (pol β protein) and therefore may utilize pol θ as a short-patch BER polymerase [57]. Pol θ catalyzes removal of 5′-dRP (Figure 3a) by an intrinsic lyase activity [58,59], a reaction for which pol β is highly specialized due to its N-terminal lyase-specific domain. Removal of these 5′-fragments must precede ligation during short-patch BER. Together, these data establish polymerase and lyase activities as vulnerabilities by which synthetic lethality could arise due to *POLQ* knockout in BER mutant backgrounds.

**Figure 4:**
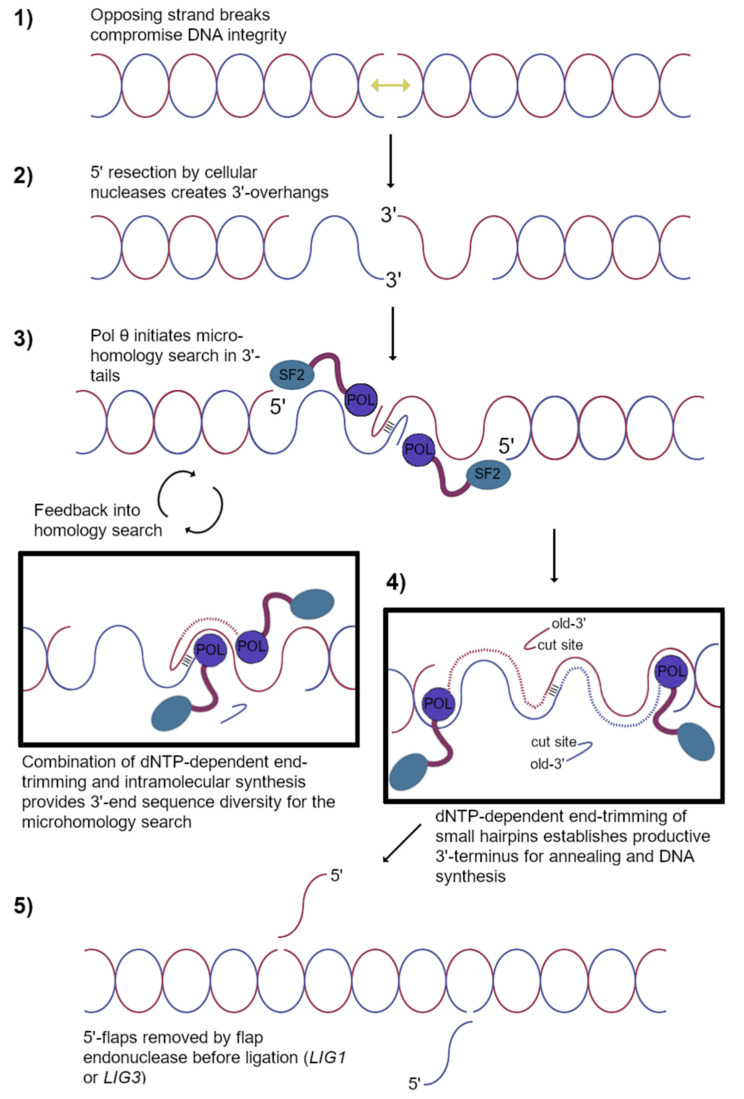
The repair of DSBs by the TMEJ pathway depends critically on pol θ to provide dNTP-dependent 3′-end trimming and template-dependent DNA synthesis activities at appropriate microhomologies.

In vitro studies aiming to distinguish the optimal mechanism of actions for *Polθ*-inhibition (Polθ-i) necessitate multiple compromises because there currently exists no encompassing recapitulation of TMEJ (Figure 4). Multi-enzyme model systems have been indispensable in informing other areas of DNA repair (NER [60], HR [6,7], BER [61]). Pol θ is unique in terms of its domain organization, but the human genome encodes many other ATPases, DNA helicases, DNA polymerases, or DNA nucleases that could conceivably compensate under selective pressure of Polθ-i. For this reason, the field has rushed to define an enzymatic activity uniquely attributed to pol θ and essential for TMEJ. Pol θ can utilize RNA as the templating strand in vitro, although the extent to which this process occurs in cells is unclear [62]. Pol θ can anneal 5′-recessed substrates with complementarity in the 3′-tail [63], but this approach only describes TMEJ in the rare case that the microhomology occupies the extreme 3′-terminus [64]. A tenacious annealing capability allows pol θ to pair low-complementary strands and extend the 3′-terminus (Figure 3b); pol θ also performs intramolecular snap-back synthesis (Figure 3d) by establishing a short hairpin configuration in its substrates [65]. DNA synthesis is therefore possible from a large variety of unusual DNA polymerase substrates, including short ssDNA oligonucleotides [66]. By all indications, pol θ specializes in template-dependent DNA synthesis [32,65,67] that is loosely primed by a microhomology, and this enzymatic activity is critical for TMEJ in cells (Figure 4).

Pol θ must conduct a homology search to align microhomologies embedded in the 3′-tails of 5′-resected substrates [64]. Although nuclease participation was predicted as a step coordinated by TMEJ, no obvious accessory candidate that could cleave a 3′-tail was identified by genetic or proteomics screens. FEN1, which prefers 5′ overhangs, does not likely function at this upstream decision point of TMEJ [33]. How pol θ accesses internal microhomologies and primes them for DNA synthesis recently came to light due to a thoroughly unanticipated result: pol θ harbors a rapid catalytic end-trimming activity (Figure 3e) intrinsic to its polymerase active site [65]. The mechanism involves the same metal-ion coordinating amino acids comprising the highly conserved polymerase motifs, which once reconfigured for end-trimming, participate in dNTP-dependent endonucleolytic cleavage specific for the 3′-end. A subsequent switch to DNA polymerase mode allows pol θ to extend the nascent 3′-end at the microhomology site immediately, bypassing the need to shuttle a weakly annealed substrate to an accessory nuclease. Polymerases and endonucleases both employ similar chemical mechanisms based on the coordination of two or three active site metal ions [68], establishing a rationale for this adaptation specific to pol θ. Never has an enzyme been shown to interconvert between two related DNA-modifying activities in the same reaction center, and this finding has profound implications for targeting a nonredundant enzymatic activity unique to TMEJ. Inhibiting the dNTP-dependent end-trimming activity of pol θ could prove particularly efficacious as a mechanism of action for small-molecule inhibitors causing few off-target artifacts. 

Endonuclease function by pol θ not only requires metal ions, but also nucleotides and an intact 3′-OH terminus on the DNA substrate. Pol θ cannot perform end-trimming of dideoxy-terminated DNA or in the presence of 2′,3′-dideoxy-nucleotides [65]. In the DNA sequence contexts evaluated to date, ddCTP inhibits end-trimming when trace amounts of dGTP are supplied in the same reaction [65]. More mechanistic studies will be required to address the underlying reason for these substrate dependencies, but the hypothesis that chemical derivatives of ddCTP might serve as competitive inhibitors of end-trimming in cells could readily be tested. Gemcitabine triphosphate represents a perfect candidate compound due to its structural similarity to ddCTP [65]. To the advantage of current Polθ-i programs, inhibitors of a template-dependent polymerase function would likely block end-trimming because the active site for both reactions are the same. Exonuclease inactivation of pol θ should be considered as a mechanism of action deactivating TMEJ in cells treated with Polθ-i.

## 7. First-in-Class Inhibitors of Pol θ Enzymatic Functions

The antimicrobial novobiocin (NVB) inhibits bacterial cells by interfering with DNA gyrase, an enzyme essential for ATP-dependent negative supercoiling of the circular bacterial genome. A screen for small molecules active against the DNA-dependent ATPase activity of the pol θ N-terminal domain demonstrates that NVB also inhibits human pol θ [26]. NVB treatment was found to abort pol θ recruitment to laser stripes, and the localization of pol θ to damaged DNA appears dependent on its ATPase activity.ATPase inhibitors would therefore be predicted to block recruitment of pol θ to DSBs in human cells. This study is the first to ascribe the localization function to the ATPase active site in vertebrates by studying the K121M variant (Figure 1), and it will be interesting to observe other labs reproducing this finding with additional approaches. *BRCA1*-deficient RPE1 cells appear highly sensitive to PARP-inhibitors (PARP-i) such as Rucaparib in clonogenic survival assays. NVB (100 µM) was shown to synergize with PARP-i in this assay, reducing the IC_50_ of Rucaparib to pM levels, and establishing proof-of-principle that Polθ-i may interact favorably with PARP-i in patients. A secondary objective for Polθ-i will be to prevent accumulated resistance to PARP-i in tumor cells [23], often mediated by selective reactivation of *BRCA2* or down-regulation of *53BP1* or its effector complexes [69]. Notably, this report of verified synergy between PARP-i and Polθ-i challenges the prior assumption that TMEJ is a PARP-dependent process [24]. If TMEJ indeed depended upon PARP-activity, then PARP-i would inhibit TMEJ de novo, and no additional sensitivity would be predicted in cells treated with Polθ-i combination therapy. Instead, evidence supports the hypothesis that the pol θ dependent TMEJ pathway plays an important role in repairing PARP-trapped lesions [70] in *BRCA*-deficient cells, indicating that TMEJ is active in the presence of PARP-i.

The use of NVB as a Polθ-i does not come without caveats. The high concentration of antibiotic needed to observe killing in RPE1 *BRCA1*-deficient cells brings into question the specificity of this drug for pol θ. NVB has a prior track-record for inhibiting other ATPases, leaving open the potential for off-target events. NVB is a known inhibitor of molecular chaperones [71], whose inactivation could have wide-spread and unpredictable consequences in cells. Nevertheless, NVB reduces the mass of tumors in both a genetically engineered mouse model (GEMM) of *BRCA1* deficiency, and in human ovarian cancer xenographs (*FANCF*-deficient TOV21G cells) as monotherapy. NVB has been the subject of prior clinical cancer research, but not extensively in the context of HR-deficiency [26]. The biotech Ideaya Bioscience (San Francisco, CA) has announced that Polθ-i Investigational New Drug (IND) trials will begin soon (https://www.ideayabio.com/pipeline/, accessed on 23 August 2021) [72], in which compounds potentially related to NVB will be tested specifically in human HR-deficient cancer patients for the first time.

**Figure 5:**
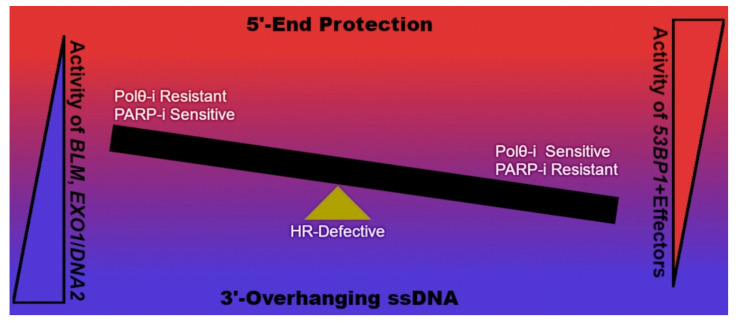
High sensitivity to Polθ-i in HR-defective cells depends on enzymatic generation of extensive 3′-overhanging ssDNA by processive exonucleases. Inhibitors of pol θ may obstruct acquired resistance to PARP-i in cancer patients with *BRCA*-gene mutations by thwarting down-regulation of *53BP1* or its effector complexes known to inhibit 5′-end resection.

Inhibitors of the pol θ polymerase domain will also be arriving in clinics shortly. Artios Pharma (Cambridge, UK) will be initiating IND on their first-in-class compounds (https://www.artiospharma.com/science/#pipeline accessed 23 August 2021), likely targeting the template-dependent DNA polymerase activity of pol θ [72]. A contribution from Zatreanu and colleagues reports on a series of compounds demonstrating high potency against DNA synthesis by pol θ in vitro [27]. These inhibitors do not compete with nucleotides at the pol-active site. Instead, kinetic analysis of ART558 reveals non-competitive inhibition in terms of nucleotide utilization and uncompetitive inhibition relating to DNA-binding, pointing to an allosteric mechanism of action. Unlike NVB, ART558 enhances recruitment of fluorescently tagged pol θ to sites of laser irradiation, suggesting that the enzyme becomes trapped at sites of DNA damage. ART558 also inhibits TMEJ specifically in a reporter assay of DSB-repair. ART558 induces γH2AX, chromosomal aberrations and micronuclei in *BRCA2*-deficient DLD1 cells, but not the parental line proficient for HR. The authors perform siRNA screens in RPE1 *BRCA1*-deficient cells, measuring the relative change in sensitivity to PARP-i and ART558 due to knockdown of numerous gene products. The transient depletion of *SHLD2* or *REV7* appear sensitizing to ART558 treatment but not to PARP-i. Because the shieldin complex functions as an effector of *53BP1* and negative regulator of 5′-end resection by nucleases [17], 3′-overhanging DNA appears critical for the mechanism of action of ART558 in cells (Figure 5). The study turns to *BRCA1^d11^* MEF cells rescued by *53BP1* [13]. In this model, siRNA targeting *Exo1*, *Dna2*, or *Blm* ease sensitivity to ART558, which is consistent with the idea that ART558 requires extensive ssDNA for potency because these genes encode enzymes critical for long-range 5′-end resection at DNA DSBs [5]. Down-regulation of *53BP1* or effectors such as *RIF1* or the shieldin complex may provide important routes to PARP-i resistance in tumor cells [69]. In cases where Polθ-i guards against these genetic alterations, drugs such as ART558 could offer more effective implementation of PARP-i therapy by preventing resistance mechanisms in cancer patients with HR-deficient disease (Figure 5).

## 8. Conclusions

Pol θ contributes to genomic stability by repairing DSBs, the rate of which becomes elevated by genetic defects in HR, C-NHEJ, or BER. The full-length pol θ protein harbors DNA-dependent ATPase, template-dependent DNA polymerase, dNTP-dependent endonuclease, and 5′-dRP lyase functions. Pol θ inhibitors that concomitantly block polymerase and endonuclease functions could prove especially potent in cancer patients because TMEJ depends critically on these enzymatic activities at multiple points in the DNA repair pathway. First-in-class inhibitors of DNA polymerase θ are now ready for evaluation in humans, with a promising foundation in basic research justifying this advance. Successful clinical trials followed by FDA approval of Polθ-i will bring hope to cancer patients with HR-deficient disease and to those patients that have acquired PARP-i resistance, a serious obstacle for this promising and newly developed class of therapeutics.

## Data Availability

Not Applicable.
